# Structure, mechanism and clinical relevance of *NRG1* fusions in cancer

**DOI:** 10.1186/s12929-026-01242-1

**Published:** 2026-04-16

**Authors:** Clarisse Thiollier-Schmitt, Manon Barre, Marie Issenmann, Emeline Cros-Perrial, Michaël Duruisseaux, Lars Petter Jordheim

**Affiliations:** 1 Université Claude Bernard Lyon 1, INSERM U-1052, CNRS 5286, Centre Léon Bérard, Centre de Recherche en Cancérologie de Lyon, 69008 Lyon, France; 2 Hospices Civils de Lyon Cancer Institute, Lyon, France

**Keywords:** NRG1, Cancer, Gene fusion, Patients, Cells

## Abstract

*NRG1* gene fusions have been identified in patients with various solid tumors. Such patients have aggressive tumors and respond poorly to currently available treatments. There are many gene partners described for *NRG1* fusions, and the structures and roles of the chimeric proteins are not always correctly understood. Here, we gather information on 665 reported patients with a total of 115 different known gene partners, and discuss structural elements supposed to play important roles in the biology of *NRG1* fusions in cancer. This gives a better understanding of the biology of *NRG1* fusions, and will help in the development of new therapeutic approaches. Our methodology described in this review can also be used for the study of other genes with disease-related fusions.

**Supplementary Information:**

The online version contains supplementary material available at 10.1186/s12929-026-01242-1.

## Introduction

**Fig. 1:**
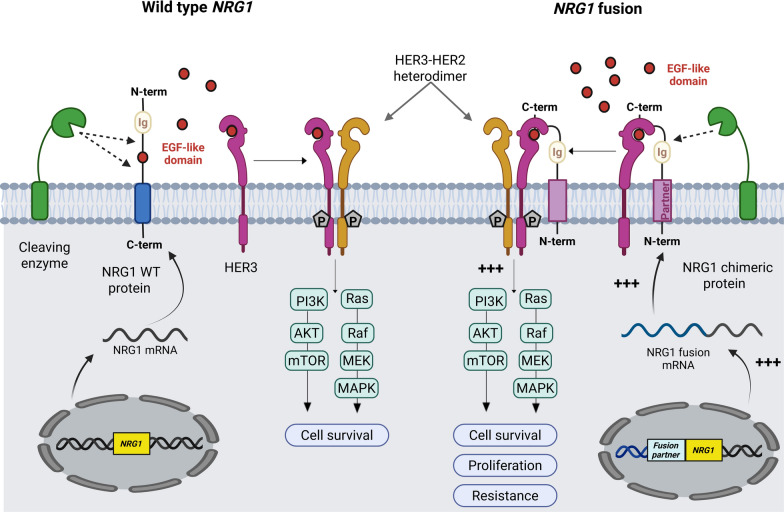
Gene and protein expression, protein cleavage, interaction with receptors and intracellular signaling of wild type *NRG1* (left) and *NRG1* fusions (right). Wild type *NRG1* corresponds to the wild type sequence of the *NRG1* gene but does not inform on the expression level. For clarity, only HER3 is indicated as NRG1 receptor, but this can also be HER4. Figure prepared with biorender.com

The *NRG1* gene is located on chromosome 8 and has a very complex structure associated with several potential transcripts and isoforms [1]. Most of them possess the three characteristic domains being an immunoglobulin-like domain, an EGF-like domain and a transmembrane domain followed by a cytoplasmic part (Table S1). The EGF-like domain of NRG1, encoded by exons 6 and 7, confers the biological activity through its binding to HER3 [2] or HER4 [3] inducing subsequent heterodimerization between HER2 and HER3 or HER4, and intracellular signaling (Fig. 1). This is possible either through direct binding of this domain still attached to the protein, or after its shedding. This latter process involves the proteases ADAM10, ADAM17 and the β-secretase BACE1 for cleavage in the extracellular domain [4–6], or the γ-secretase or signal peptide peptidases SSPL2a and SSPL2b for cleavage in transmembrane domains [7, 8].

As detailed here after, *NRG1* is described in a large variety of gene fusions with at least 115 gene partners and involving various sites of *NRG1*. What seems to be a general concept, although not always the case, is that the chimeric protein contains the N-terminal part of the partner protein and the C-terminal part of NRG1. As the EGF-like domain should be present to enable binding to the receptor, the majority of the pathology-related fusion genes start with exon 6 for NRG1.

The first identification of an *NRG1*-related gene fusion was published in 1999 and concerned the *DOC4(TENM4)*-*NRG1* fusion in MDA-MB-175 breast cancer cells [9]. The presence of an abnormal variant of NRG1 in this cell line had been suspected some years earlier [10] and was much later shown to be *PPP6R3-TENM4-NRG1* [11]. The resulting gene is transcribed to a higher level than wild type *NRG1*, due to the regulation by the *DOC4* promoter [12]. Later, a study on a series of breast and pancreatic cancer cell lines confirmed that *NRG1* represents a fragile site for both translocations and gains [13]. As cancer cell lines derive from patients, this could either indicate the presence of such modifications in pathological settings, or that the in vitro culture of the cells could induce such variations. Today, several cell lines expressing *NRG1* fusions exist. These either grow as patient-derived cancer cell lines or are obtained by transfection of cancer cells with expression vectors encoding the chimeric proteins.

Despite the continuous publication of reviews on NRG1 [14–28], we propose a thorough overview of the literature, with a particular emphasis on gene fusions involving *NRG1* and an original discussion about the underlying mechanisms. We believe that this approach could be applied to other gene fusions in cancer and other diseases.

## *NRG1* fusions in cancer patients

A first report on *NRG1* rearrangements in clinical samples came in 2005 with the study of 438 breast cancer samples [29]. Out of 358 analyzable cases, 17 (4.7%) showed either increased copy number of 3’ as compared to the 5’ (12 cases), increased 5’ as compared to 3’ (2 cases) or amplification of both 3’ and 5’ (3 cases). Since 2014, a large amount of work has reported the presence of *NRG1* fusions in patients with solid tumors, with a preponderance of lung cancers (Table S2). These are either case reports with a limited number of patients, or larger series of one specific or several cancer types.

We performed a search for clinical cases of *NRG1* fusions. We used the terms “*NRG1* fusion” and “patient sample” on PubMed and reviewed all original papers and reviews to identify cases and additional references. Databases were not directly interrogated but sometimes used in the included publications. We referenced fusion partner, tumor types and details on the sequence of the fusion, and included all samples (missing information was indicated as “unknown”). We identified a total of 42 relevant reports between the first publication in 2014 and May 2025. Some publications reported on samples from public databases (TGCA, MSK-IMPACT…) without clear identification for each sample. Thus, we decided to include these cases from all reports, inducing a very limited risk for counting samples twice.

**Fig. 2:**
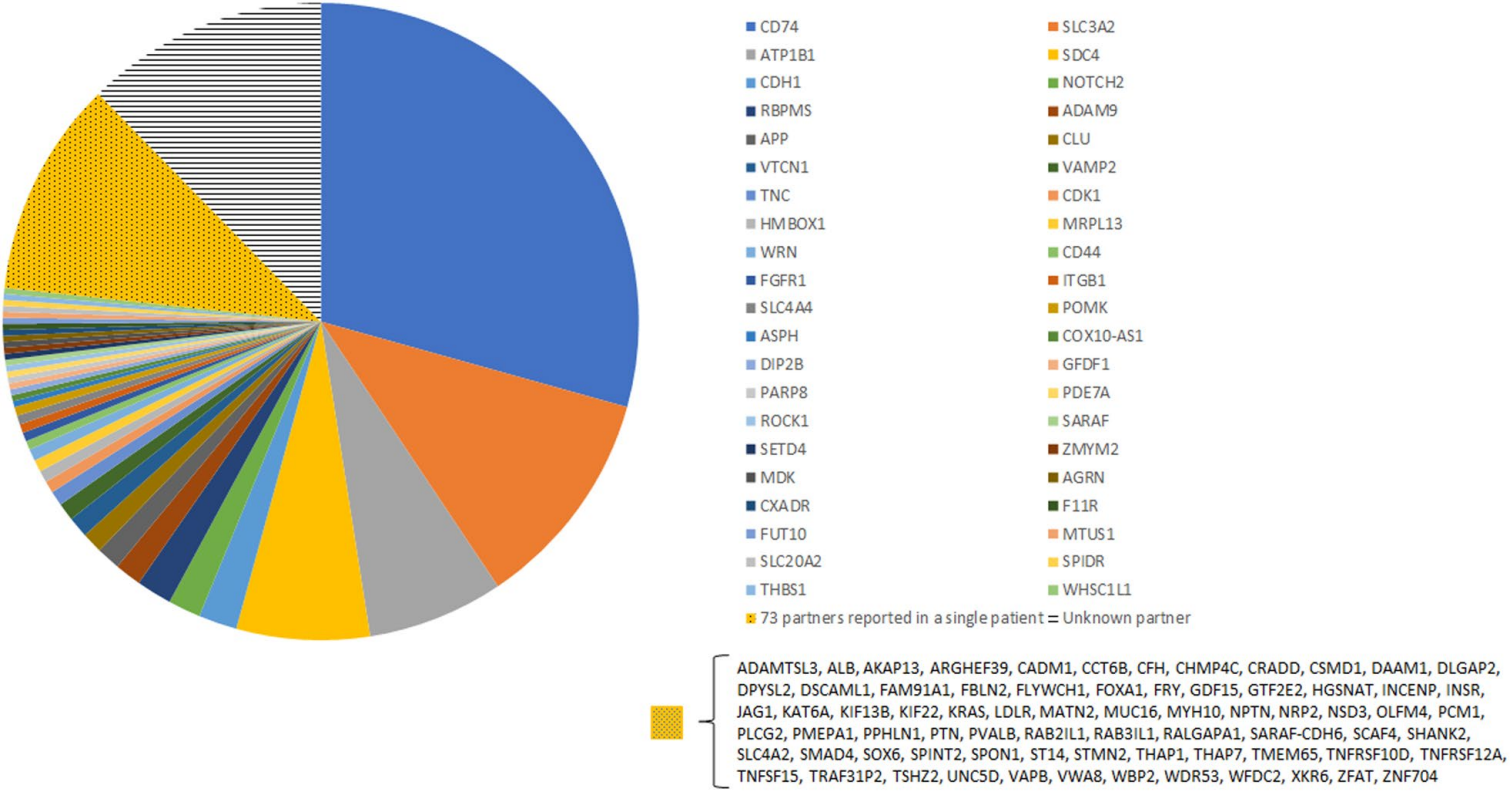
Distribution of the partner genes in the 665 reported cancer patients. The 73 unique cases are clustered together, and the 82 unknown partner genes appear in one group. Details of cases are in Table S2

**Fig. 3:**
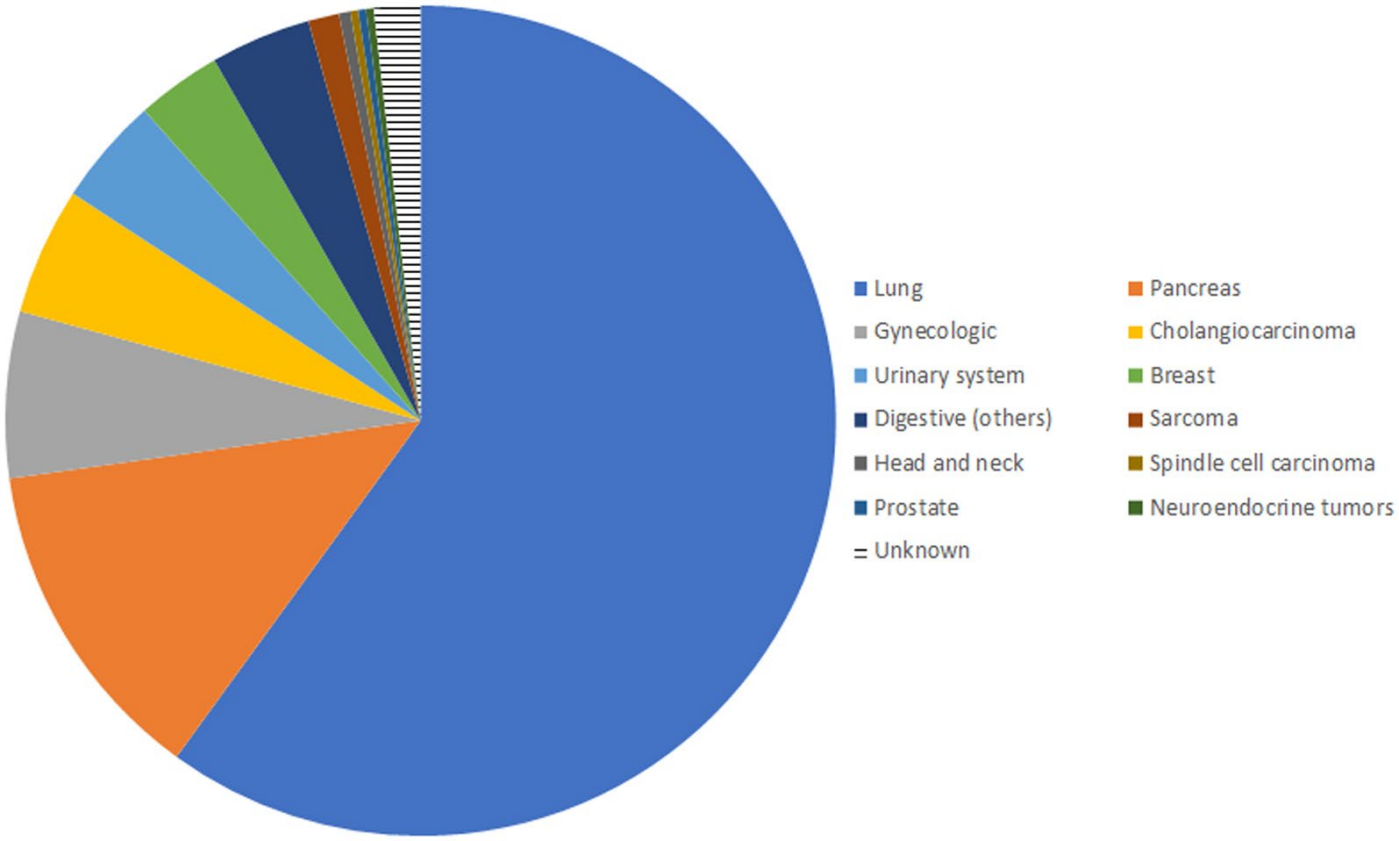
Organ distribution of the 665 reported cancer patients with *NRG1* fusions. Twelve cases (1.8%) are not reported with their localization. Details of cases are in Table S2

We identified a total of 665 patients with *NRG1* fusions. For 583 of them, the partner gene was known and represented 115 different genes, whereas for the 82 remaining patients, the partner gene was not specified (Fig. 2). A third of the known partner genes (42/115 = 36.5%) are described for two or more patients accounting for 87.5% of the patients with known partners (510/583), whereas the remaining 73 partner genes are only reported once. The most frequent known partner genes are *CD74* (195/665 = 29.3%), *SLC3A2* (75/665 = 11.3%), *ATP1B1* (46/665 = 6.9%) and *SDC4* (45/665 = 6.8%). The vast majority of cases are from patients with lung cancer (399/665 = 60.0%), followed by pancreatic cancer (85/665 = 12.8%) and gynecologic cancers (ovarian, fallopian tubes and primary peritoneal carcinomas, uterine carcinosarcoma, endometrial sarcoma and spindle cell carcinoma in uterus) (43/665 = 6.5%) (Fig. 3). Only one out of the 665 cases was clearly reported as being from a pediatric patient, a 16 years old girl with cholangiocarcinoma with the fusion *AGRN*-*NRG1* x [30] (this nomenclature gives information on the fusion, this one includes exons of *AGRN* upstream of exon 2 (exon 2 included) and exons of *NRG1* downstream of exon 2 (exon 2 included)).

The partner genes differ according to the localization of the tumor (Figure S1). In lung cancer, 34 partners were identified among the 234 patients with a described partner gene, out of which 15 were seen in at least 2 patients (Figure S1A). The most prevalent gene partners were *CD74* (114/234 = 48.7%) and *SLC3A2* (44/234 = 18.8%). In pancreatic cancer, 12 partner genes were identified among the 32 patients with a described partner, with a high prevalence of *ATP1B1* (15/32 = 46.9%) (Figure S1B). In the 43 cases of gynecological origin, there was a very high variability as 31 different partners have been described for the 39 patients with known partner genes (Figure S1C). This high variability was also seen in urinary cancers (14 known partners for 20 cases with a known partner, Figure S1D), and in the 11 breast cancer cases with 10 different partner genes (Figure S1E).

When looking at the organ distribution of the eight main partner genes of *NRG1*, it appears clear that *CD74* (114/118 = 96.6%) and *SLC3A2* (44/46 = 95.7%) are almost exclusively expressed in the lung (Figure S2A and B). Interestingly, *CD74* has also been described as a fusion partner *ROS1*, *NTRK1* and *NRG2α* in lung cancer, suggesting an important role of this gene in lung cancer biology [32]. *SDC4* (Figure S2C) and *ATP1B1* (Figure S2D) are less specific although there is a preference for *SDC4* for lung cancer (24/32 = 75.0%) and for *ATP1B1* for pancreatic cancer (15/23 = 65.2%), whereas *CLU* is only reported in ovary cancers (Figure S2E, 7/7 = 100%). Some examples of less organ-specific genes are *ADAM9* (Figure S2F, 9 fusions found in 6 different organs), *CDH1* (Figure S2G, 9 fusions found in 4 different organs) and *NOTCH2* (Figure S2H, 7 fusions found in 5 different organs).

Finally, using clearly described data from selected publications, we have calculated the epidemiological frequencies of *NRG1* fusions to be 0.22% (0.14–0.72%) in lung adenocarcinoma (186 cases in 85,338 patients) [33–41], 0.15% (0.00–0.44%) in ovarian cancers (31 cases in 20,820 patients) [33, 36, 40, 42], 0.12% (0.04–0.70%) in breast cancers (15 cases in 12,447 patients) [11, 33, 35, 36, 40, 41] and 0.06% (0.02–0.48%) in pancreatic cancers (11 cases in 17,939 patients) [33, 35, 36, 40, 41]. It is important to notice that these values do not take into account cancer subtypes (driver mutations, histological particularities…) and ethnical variabilities, that can explain some of the important ranges for the frequencies.

## Mechanistic explanation of the aggressiveness of *NRG1* fusion positive tumors

The mechanism by which NRG1 stimulates cell proliferation and subsequently cellular aggressiveness and resistance to therapy, is overall well described and understood [43–50]. Briefly, this comprises the expression of NRG1 at the cellular membrane, probably the proteolytic cleavage of the extracellular part liberating the EGF-like domain, the binding of this latter to HER3 or HER4 on the same or a nearby cell, the induction of their heterodimerization with HER2 as well as subsequent phosphorylation cascades and intracellular signaling. However, in the case of *NRG1* fusions, several questions are only partially answered, and the precise mechanism remains unknown.

### Why fuse?

**Fig. 4:**
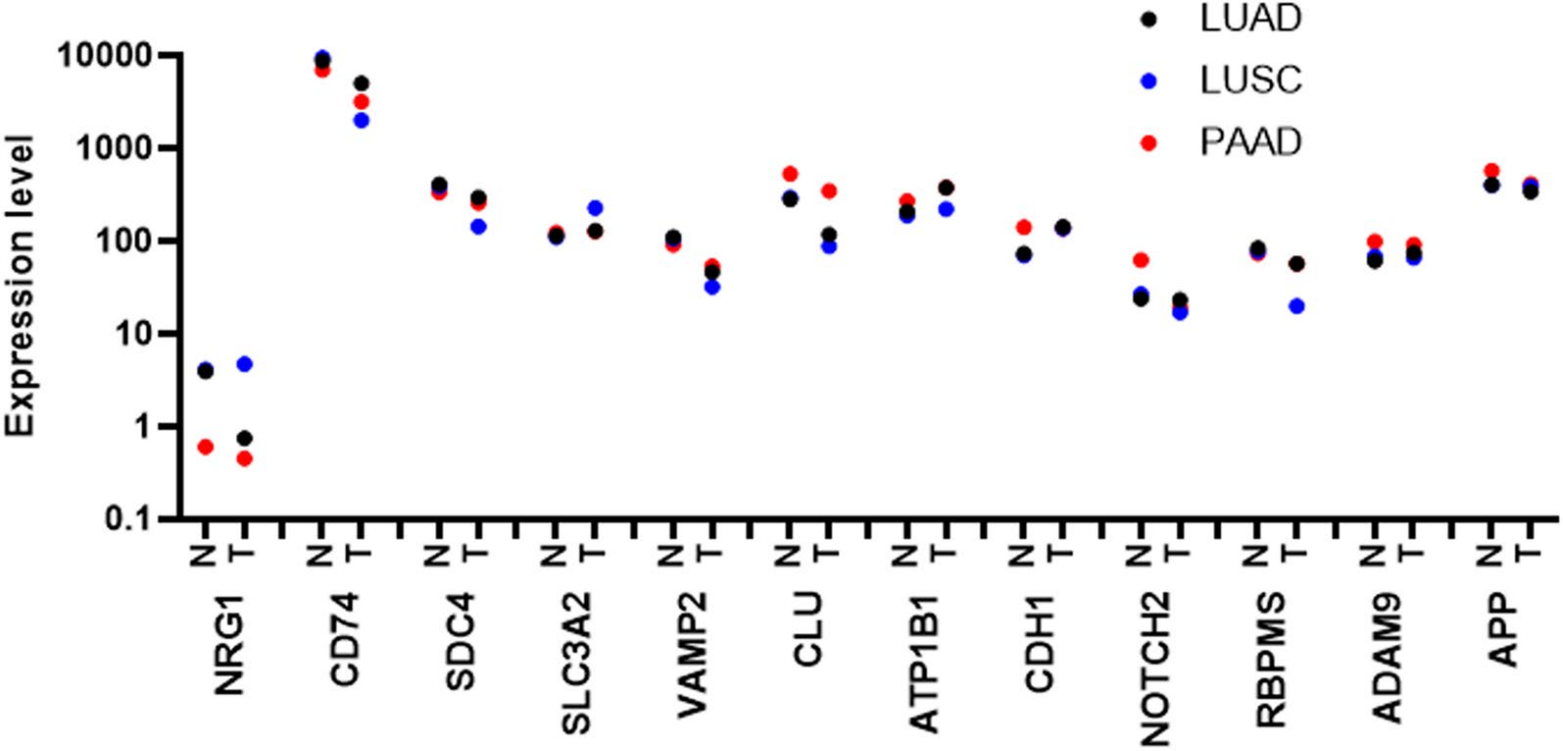
mRNA expression of *NRG1* and main fusion partners in normal tissue from lung and pancreas as well as in lung adenocarcinoma (LUAD), lung squamous cell carcinoma (LUSC) and pancreatic adenocarcinoma (PAAD). Expressions are for wild-type genes and not in a context of *NRG1* fusions and are median values of transcripts per million from the TCGA and extracted from the UALCAN website. The sizes of the cohorts are: LUAD = 59 normal and 515 tumoral, LUSC = 52 normal and 503 tumoral, PAAD = 4 normal and 178 tumoral. N: normal, T: tumoral

First of all, there is the question about why *NRG1* fuses to other genes in order to give the cells a proliferative advantage. As *NRG1* fusion containing lung tumors are described as having less tumor mutational burden than other defined lung cancers, the fusions are not expected to result from an overall increase in mutation rates [51]. As shown in Fig. 4 containing data from TCGA databases, wild-type *NRG1* has a very low expression in various healthy organs and in tumor tissues (lung and pancreas), whereas 11 fusion partners are up to 7000-fold more expressed (*e.g. CD74* in PAAD samples). This suggests that the fusions between the promoter of the fusion partner and the C-terminal part of *NRG1* induces an important increase of the biologically relevant EGF-like domain in the extracellular media. We hypothesize that this can occur in healthy or preneoplastic cells undergoing particular stress inducing chromosomal instability, for example through altered mitosis or loss of cell cycle check points. Various chromosomal modifications will take place in many cells, but only some, such as discussed *NRG1* fusions, will give tremendous proliferative advantage and allow cells to develop into a more or less clonal tumor. Our promotor-related hypothesis is strengthened by the observation of the fusion between *WRN* and *NRG1* that produces a wild type NRG1 protein with an increased expression due to the transcriptional regulation by the *WRN* promoter [34]. In this study it was shown that all samples with *NRG1* fusions had high NRG1 expression as compared to samples with wild-type NRG1, identifying them as outlier values. However, the same was observed for a series of samples without *NRG1* fusions or any other known driver aberration, suggesting that other mechanisms such as epigenetic regulations can also increase NRG1 expression and promote tumor cell growth. Outlier NRG1-expression in *NRG1* fusion positive samples was confirmed in another study, although in a lung- and pancreas-specific manner [35]. Indeed, NRG1 fusion positive samples in other cancer types, in particular head and neck carcinoma and uterine carcinosarcoma, showed a normal expression level of NRG1. A patient with intrahepatic cholangiocarcinoma also showed a high NRG1 expression from an *ATP1B1*-*NRG1* fusion as compared to data from TCGA, whereas a *SDC4*-*NRG1* fusion was not associated with a high expression [52]. An additional observation that underlies our promoter-related hypothesis, is that *ROS1* shares several fusion partners with *NRG1* in lung cancer [53]. As the identified fusion partners are not necessarily the highest expressed genes in the tumoral tissues, other reasons, such as space proximity and sequences, are supposed to be relevant as well.

### To cleave or not to cleave

**Fig. 5:**
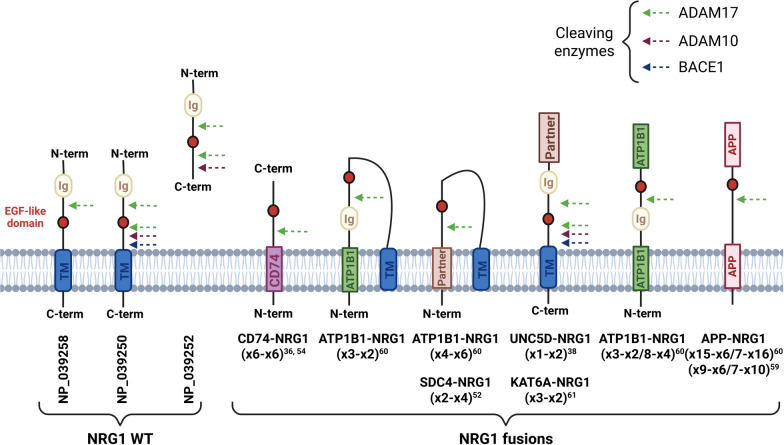
Predicted schematic structures of sufficiently described chimeric proteins as well as the three corresponding isoforms of wild type NRG1 proteins (NP_039258, NP_0039250 and NP_039252). Cleavage sites for ADAM10 (purple arrow), ADAM17 (green arrow) and BACE1 (blue arrow) are indicated in order to suggest the possibility of accumulating free EGF-like domain in the extracellular media with the different fusions. Indicated *NRG1* fusions are discussed in the text. Figure prepared with biorender.com

Another question concerns the proteolytic cleavage of the extracellular domain of chimeric proteins, and the prerequisite of this for biological activity. As mentioned earlier, proteases can cleave wild type NRG1 on both sides of the EGF-like domain or in the transmembrane domains. Depending on the expressed isoform of NRG1 and the proximity of HER3 or HER4, one or two digestions might be necessary for the EGF-like domain to bind to its receptors. In many reports of *NRG1* fusions in cancer patients, the reference NRG1 protein is NP_039252 of 241 amino acids containing the Ig domain and the EGF-like domain but no transmembrane domain (Fig. 5). If this is true, the fusion partner should necessarily bring a transmembrane domain in order to express the EGF-like domain outside the cell. Concerning the *CD74*-fusions, the transmembrane domain is contained in exon 2 [32] which is always present as fusions occur after exons 5, 6, 7 or 8, at least in samples with known sequences. For example, in a chimeric protein obtained from the fusion between the first 6 exons of *CD74* and exons 6 and 7 of *NRG1* (x6-x6) [36, 54], the EGF-like domain will be expressed outside the cell and contain the cleavage site for ADAM17 between CD74 and the EGF-like domain (Fig. 5). The correct expression of such a chimeric protein was confirmed in NIH3T3 and NCI-H2052 cells, showing the presence of CD74 only inside the cells and of NRG1 outside the cells [54]. This was also seen with *SLC3A2*-*NRG1* (x5-x6), *VAMP2*-*NRG1* (x4-x4) and *DOC4*-*NRG1* (x12-x2) but not with *CD74*-*NRG1* (x8-x6), *SDC4*-*NRG1* (x2-x6), *RBPMS*-*NRG1* (x5-x2), *ATP1B1*-*NRG1* (x3-x2) and *CLU*-*NRG1* (x2-x6) fusions expressed in the IL3-dependent pro-B cell line Ba/F3 [55]. Using a similar chimeric protein with the transmembrane domain of SLC3A2 and the C-terminal of NRG1 expressed in HEK-293T cells, it was shown that the protumoral activity was promoted by the supernatant of the cultured cells [56]. The same authors later showed that the stimulating effect was inhibited by the ADAM17 inhibitor GM6001 [57], indicating that at least in this case, the proteolytic release of the EGF-like domain is needed for the biological activity of the chimeric proteins. Although *NRG1* fusions are often described as being mutually exclusive with other oncogenic driver mutations such as in *KRAS*, *KRAS* mutations enhanced the oncogenic properties of *NRG1* fusions by increasing the expression of ADAM17 [57]. Moreover, Schram *et al.* reported 10 cases, among a cohort of 204 *NRG1* fusion positive patients, with a concomitant oncogenic driver, including *KRAS* mutation (G12D, G12C, G12A, G12V, K117N) and *MET* amplification [58], supporting the possibility of co-existence between an *NRG1* fusion and other oncogenic drivers.

Another reference sequence for NRG1 was used by Jones MR *et al.* when identifying *SDC4* and *ATP1B1* fusions, this probably being the 640 amino-acids long NP_039258 [52]. In that case, the protein only contains one ADAM17 cleavage site, as well as the transmembrane domain of NRG1 (Fig. 5). Cleavage can also occur within the transmembrane domain by γ-secretase, although this is supposed to take place after initial cleavage by BACE1 [7]. Particular chimeric proteins are produced in pancreatic cancers by the insertion of the EGF-like domain within the APP protein [59, 60]. These proteins have transmembrane domains from APP, and the NRG1-part is likely to contain at least one ADAM17 cleavage site. Two other chimeric proteins were reported in pancreatic cancer with *ATP1B1*, either with 4 exons fused to exons 6–13 of *NRG1*, or exons 2–8 of *NRG1* inserted between exons 3 and 4 of *ATP1B1* [60]. However, whether exons 8–13 were expressed in the first case here was not evident from the RNA sequencing data presented. In a study reporting on 41 cases of *NRG1* fusions, several chimeric proteins were predicted not to have any transmembrane domain or signal peptide, such as *COX10*-*NRG1*, *POMK*-*NRG1* or *WHSC1L1*-*NRG1* [36]. The *UNC5D*-*NRG1* fusion (x1-x2) was definitely described with the sequence NP_039250 and does contain the transmembrane domain of NRG1, and thus the γ-secretase cleavage site, in addition to a signal peptide from UNC5D [38]. Reported in the same paper, it is difficult to predict where and in what form *TNC*-*NRG1* is expressed as a chimeric protein. However, the *KAT6A*-*NRG1* fusion (x3-x6) found in a patient with renal cell carcinoma, is also reported with the transmembrane domain of *NRG1* [61]. For three fusions (*PPHLN1*-*NRG1*, *HMBOX1*-*NRG1* and *MTUS1*-*NRG1*) described in sarcomas, the partner genes only brought the 5’-UTR together with the promoter activity [62]. If the predicted chimeric proteins presented are correct (using NP_039252 as reference for NRG1), it is again difficult to understand how the EGF-like domains from these proteins would be secreted in the extracellular media if this is needed for biological activity.

### Upstream or downstream

The next question concerns the dependency on the membrane expression of the chimeric protein, and the extracellular expression of the EGF-like domain, supposed to carry the biological activity. The large majority of reported *NRG1* fusions have the fusion partner at the 5’ and *NRG1* at the 3’. This implies the promoter activity of the partner and the expression of the EGF-like domain of NRG1 in the extracellular environment, due to the transmembrane domain either of the partner gene or of NRG1. There are, however, some examples of *NRG1* fusions that occur the other way around, as for example with *CD74* or *CDH1* at 3’ and *NRG1* at 5’ [58], *NRG1* x3 fused with *STMN2* x2 and the *NRG1* x1 fused with *PMEPA1* x2, or in the correct order between *PCM1* and *NRG1* but without the EGF-like domain [35]. It is very difficult to imagine how these fusions can give any proliferative advantage to cells, or participate in the transformation process, if the EGF-like domain is needed. They might be leftovers from early initial genomic instabilities, and these cells might harbor another driver mutation.

### Are fusions enough?

Further, there is the question about the actual biological activity of chimeric proteins issued from *NRG1* fusions, and whether they are sufficient to explain the reported oncogenic properties. Several reports on cell-based research are supporting this. For example, NIH3T3 cells with induced expression of CD74-NRG1 (x6-x6) chimeric protein were able to form colonies in an anchorage-independent way, suggesting oncogenic properties [63]. This effect was inhibited by afatinib or lapatinib, confirming the mechanism of action through HER signaling. The same observation was done in NCI-H1568 cells with either *CD74*-*NRG1* [54] or *VAMP2*-*NRG1* [64] as well as in lung cancer H322 or breast cancer BT20 cells [65], and this growth was dependent on the EGF-like domain of NRG1 as a chimeric protein deleted for this domain did not have the same properties [54]. The expression of a chimeric CD74-NRG1 protein in normal lung BEAS-2B cells, increased proliferation, migration and EMT, and induced an alteration in morphology [34]. The expression of both wild type NRG1 and the chimeric protein SLC3A2-NRG1 in Calu-3, HCC827 and HCC358 cells was associated with increased migration, proliferation and in vivo tumor growth, and again this was dependent on the presence of the EGF-like domain [56]. This observation is in favor of our promotor-based hypothesis as both proteins are expected to be expressed at the same level since they are regulated by the same promotor. The oncogenic properties of *NRG1* fusions were clearly established with the transgenic mouse expressing *CD74*-*NRG1*, with a 100% of mice developing tumors before 130 days, and a shorter survival as compared to control mice [66]. The heterodimerization between HER2 and HER3 was needed for this process, and fusion-containing cells could not be further stimulated by adding soluble NRG1. Additional proof of the enhanced clinical aggressiveness of *NRG1* fusions included the observation of a better overall survival (p = 0.019), but not disease-free survival (p = 0.113) in stage I lung mucinous adenocarcinoma patients without *NRG1* fusions (n = 33) as compared to patients with *SLC3A2*-*NRG1* or *CD74*-*NRG1* fusions (n = 12) [56].

Whether *NRG1* fusions are enough to drive oncogenic properties in cases with other known oncogenic drivers such as *KRAS* mutations [57, 58] or *MET* amplification [58] is an even more intriguing question. There is, to our knowledge, no data suggesting that the *NRG1* fusion or the other modification is predominant and sufficient. This could be evaluated by the study of the sensitivity of relevant patient-derived models such as organoids to drugs targeting one or the other oncogenic driver, as well as their combination. This represents an important step in order to propose an adequate treatment to the small cohort of patients with two oncogenic drivers.

### Homogeneous or heterogeneous signaling?

Finally, it remains unknown whether different chimeric proteins have various signaling in HER3/HER4-expressing target cells. It was first shown that the chimeric CD74-NRG1 protein signals through HER2-HER3 with induction of Akt, ERK and mTOR both using patient samples and cell lines [54, 56, 63, 67]. Similar observations were done with *VAMP2-NRG1* transfected into NCI-H1568 cells [64] and *RALGAPA1*-*NRG1* in H3122 cells [68]. Finally, the *PPP6R3-TENM4-NRG1* fusions in MDA-MB-175 cells and the *SLC3A2*-*NRG1* fusion in LUAD-0061AS3 cells can also activate HER4 and EGFR signaling [55]. We believe that any differences in signaling pathways are due to the expression pattern of receptors of the EGFR-family as well as of intracellular effectors, rather than the nature of the gene fusion. This can explain the weak response rate to treatments used for *NRG1* fusion positive cancer patients (25–35%) [51, 58, 69]. Indeed, patients are given inhibitors for HER2 or HER2/HER3 because the chimeric proteins are supposed to signal through these receptors and their downstream effectors. However, there is to date no confirmation that this signaling is occurring in all patients, and there is no available data on the variability of expression of targets and effectors between patients or within their tumors. Integrated studies on all these proteins in patient samples analyzed together with clinical data and response to treatment, should allow for a better understanding of this question.

### What is an active *NRG1* fusion?

As seen in the paragraph on NRG1 fusions in cancer patients, there is a large variety in the described *NRG1* fusions in cancer patients, and from what we discuss in here over, they are probably not all active. We expect that *NRG1* fusions will be detected in an increasing proportion of patients due to the more frequent use of sequencing methods for diagnostic purposes. Therefore, it is important to define clinically relevant *NRG1* fusions, and we propose a set of criteria based on current knowledge in Table 1, that was also used in a recent clinical trial [58]. In order to decide whether or not a patient has an active *NRG1* fusion, it is important to have highly relevant methods for the detection of such genetic aberrations. The currently used methods have been recently reviewed elsewhere [14, 19, 23, 28, 70–72] and include DNA and RNA sequencing, FISH and immunohistochemistry. The technical development and validation in this field should take into consideration the points indicated in Table 1.

**Table 1:** Criteria for functionality of *NRG1* fusions based on current knowledge

High level of validation	• Presence of EGF-like domain (exons 6 + 7 of *NRG1*) • In-frame fusion between *NRG1* and partner gene
Potential importance, to be validated	• Presence of at least one cleavage site to liberate EFG-like domain in the extracellular media • Partner gene in 5’-position and *NRG1* in 3’-position • Presence of transmembrane domain in fusion partner or in *NRG1* • Absence of other oncogenic drivers • Expression of HER2, HER3 and downstream effectors in the tumor

## Therapeutic perspectives for cancer patients with NRG1 fusions

**Fig. 6:**
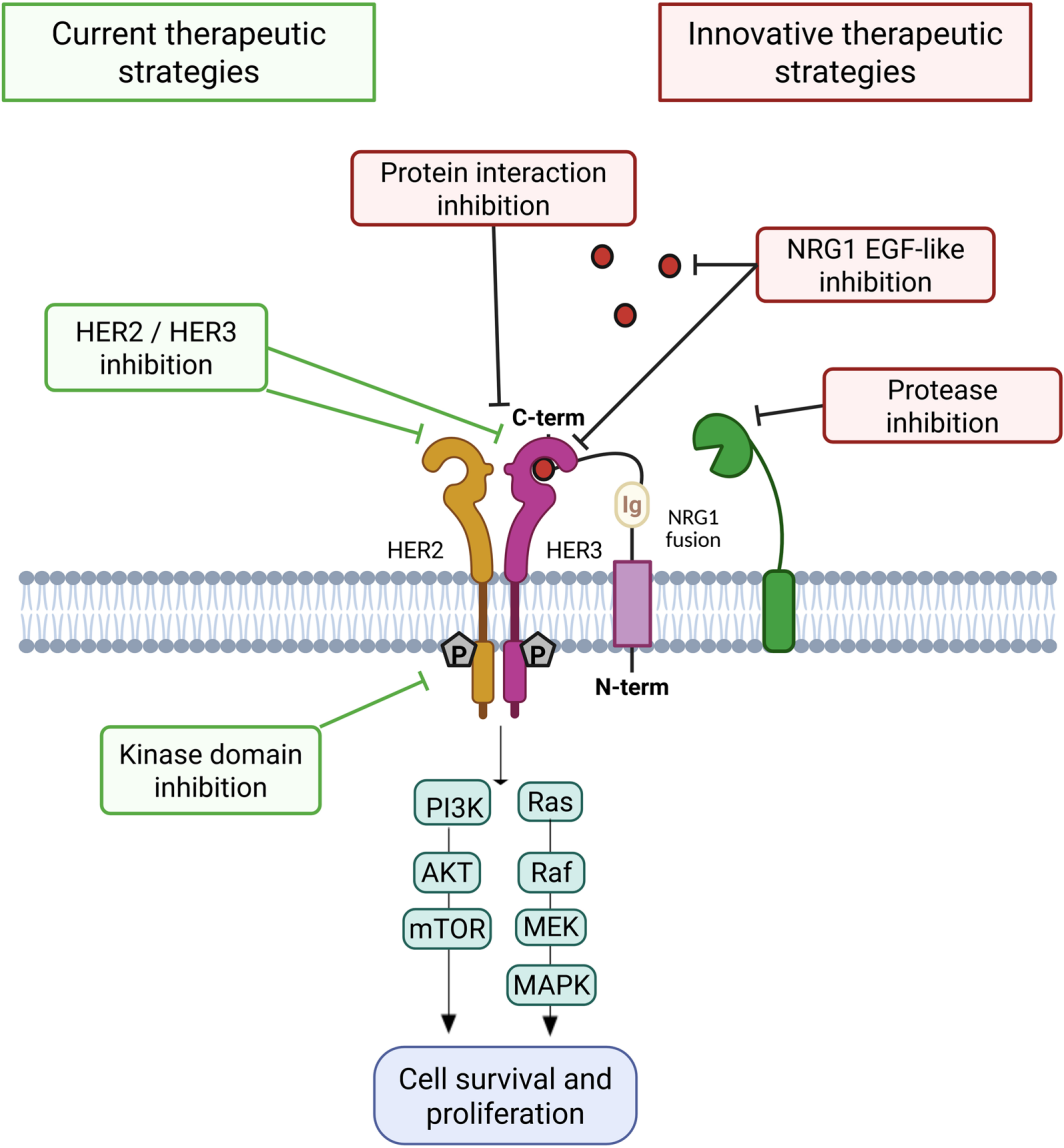
Therapeutic options for targeting the NRG1 pathway in cancer. In green: already developed approaches. In red: new approaches discussed in this review. Figure prepared with biorender.com

*NRG1* fusion positive patients have overall a poor response to classical cancer treatments [51]. The knowledge and understanding of the underlying mechanisms for the protumoral and aggressive roles of associated chimeric proteins explained and discussed in this review, constitute the bases for new therapeutic options for these patients. These are mainly based on the targeting of HER receptors, their tyrosine kinase activities or the subsequent intracellular signaling, using small molecules or antibodies (see [15, 17, 23, 28] for recent reviews). We will not describe these options again here, but discuss potential new ways to target any part of the pathway involved in triggering cancer promoting signals in *NRG1* fusion positive patients, and inspired by the current literature review (Fig. 6).

The first option, that to our knowledge has not been deeply investigated, would be the direct inhibition of NRG1, or more precisely the soluble and biologically active EGF-like domain. This could be targeted by monoclonal therapeutic antibodies, and as the target is expressed in the extracellular environment, the accessibility of the target is not a limitation. Such targeting would spare the body for the inhibition of physiological HER signaling occurring with other ligands, and thus be associated with less adverse effects. However, due to the numerous EGF-like domain-containing proteins [73], particular efforts should be made in order to be specific to NRG1. Two reports using anti-NRG1 antibodies have been published. In the first, two antibodies targeting the EGF-like domain without binding to EGF protein (YW538.24.71 and YW526.90.28), were shown to inhibit HER3 and HER4 phosphorylation and to increase the antitumoral activity of chemotherapy in mice [74]. The second report investigated an antibody targeting the Ig-domain (7E3), that was also able to block the NRG1-induced HER3 activation as well as subsequent migration and in vivo growth of pancreatic cancer cells [44]. However, no data are available about the activity of these antibodies on *NRG1* fusion models.

If the NRG1 cleavage is needed, a second option resides in the inhibition of the proteolytic cleavage of NRG1 resulting in the liberation of the EGF-like domain. This could be obtained by the direct inhibition of proteases, as exemplified in the preclinical studies on ADAM17 [57], although the systematic inhibition of such proteins could be harmful. As for example, the constitutional ADAM17 knock out mice are not viable, and conditional knock out is associated with important unwanted biological effects [75]. A way to increase the selectivity here would be to use either antibodies, peptides or aptamers that recognize the cleavage site on the NRG1-containing chimeric proteins.

A third therapeutic option consists in the inhibition of protein–protein interactions involved in the NRG1 signaling. These are multiple, and include the binding of EGF-like domain to HER3 or HER4, the interaction between HER3 or HER4 with HER2, as well as physical interaction between intracellular effector proteins. The latter option will again be unspecific, whereas the two first should have a good specificity for the NRG1-related signaling. Three antibodies (zenocutuzumab, seribantumab and HMBD-001) block the heterodimerization between HER3 and HER2. Seribantumab acts through the fixation at the NRG1-binding domain of HER3 and thus blocks all subsequent signaling [76], and gave an objective response rate of 36% in a phase 2 clinical trial with *NRG1* fusion positive patients (NCT04383210, [69, 77]). HMBD-001 binds directly to the HER2-binding interface of HER3 [78], and clinical trials are ongoing (NCT05057013, NCT05910827, NCT05919537). Zenocutuzumab binds to HER2 and thus positions the anti-HER3 arm of the antibody to block the NRG1-HER3 interaction. It was associated with a 31% response rate and a median duration of response of 16.5 months in NSCLC patients and with a 44% response rate and a median duration of response of 9.1 months in pancreatic cancer [58], and zenocutuzumab was granted accelerated approval for NSCLC and pancreatic cancers with *NRG1* fusions in December 2024.

The HER2 inhibitor afatinib has also been used in patients with *NRG1* fusions. It first showed a 25% response rate and a 2.8-month progression-free survival in NSCLC patients [51], and this increased to 37.5% and 5.5 months respectively in a study on patients with a variety of *NRG1* fusion positive solid tumors [79]. Some case reports have also been published, showing a potential interest for this drug in this clinical setting [80–83]. In a translational work on NRG1-dependent circulating tumor cells in breast cancer patients, it was shown that fibroblast growth factor 1 (FGFR1) is expressed when NRG1 is blocked, thus acting as a compensatory mechanism in cell survival [84]. Although no data exists, it could be speculated that this is also the case in *NRG1* fusion positive patients in which NRG1 signaling is blocked. A therapeutic option would then be to block both NRG1 and FGFR1 in such patients. It should be noted that *FGFR1* has been reported as a fusion partner for *NRG1* in 3 lung cancer patients [51]. Depending on the mechanism of upregulation of FGFR1 when NRG1 is blocked, a NRG1 blockage could increase the expression of the chimeric protein between FGFR1 and NRG1 through a transcriptional regulation. Finally, it has also been shown that NRG1 secreted from adipocytes in metastatic urothelial carcinoma induces resistance to the FGFR inhibitor erdafitinib, suggesting a reciprocal effect between these pathways [46].

The therapeutic approaches proposed here are all compatible with *NRG1* fusion positive patients, but also with patients expressing high levels of wild-type NRG1. As shown in earlier cited reports, some *NRG1* fusion negative patients have high NRG1 expression [34, 35]. Therefore, it is also important to identify a relevant marker for the involvement of NRG1 in the aggressiveness of tumors, in order to choose an optimal treatment option. Suggested biomarkers include the expression of the NRG1-related EGF-like domain, or HER3, HER4 or HER2 proteins. Finally, we believe that ongoing work on cell models, patient samples and within clinical trials, will molecularly describe the reasons why *NRG1* fusion cancers are difficult to treat and allow researchers to propose new therapeutic options for these patients.

## Conclusion and future perspectives

As evidenced by our review of reported clinical cases with *NRG1* fusions, these genomic modifications remain rare but present in a variety of cancers and include a variety of partner genes. Whereas some reports are very precise about the nature of the fusions, others are more restrictive in the information, sometimes even excluding the identity of the partner gene. We believe that additional efforts should be made on the identification and the description of these fusions. This will help in developing relevant models expressing the chimeric proteins in order to better understand their biological functions. Indeed, some are clearly expressed at the cellular membrane and liberating the EGF-like domain of NRG1, whereas for others, it is difficult to understand how a cell would benefit from the expression of the chimeric proteins. Such experimental work will also help in understanding the potential differences between partner genes as for example in their relative organ specificity. They will play an essential role for the validation of new therapeutic approaches that will be or not be specific to given clinical situations (organ, fusion…). We believe that ongoing and upcoming fundamental, translational and clinical research will answer several of our questions in the near future, and constitute the scientific basis for the future treatments of *NRG1* fusion positive cancer patients.

## Supplementary Information

Additional file 1.

## Data Availability

No datasets were generated or analysed during the current study.
