# Supplementary material for: Structure, mechanism and clinical relevance of NRG1 fusions in cancer
Source: J Biomed Sci. 2026 Apr 16;33:41. doi: 10.1186/s12929-026-01242-1 (PMC13085346; doi:10.1186/s12929-026-01242-1)
Supplement: Supplementary file 1 — Additional file 1. [file 12929_2026_1242_MOESM1_ESM.docx]

Supplemental information

Structure, mechanism and clinical relevance of *NRG1* fusions in cancer

Clarisse Thiollier-Schmitt, Manon Barre, Marie Issenmann, Emeline Cros-Perrial, Michaël Duruisseaux, Lars Petter Jordheim

Université Claude Bernard Lyon 1, INSERM U-1052, CNRS 5286, Centre Léon Bérard, Centre de Recherche en Cancérologie de Lyon, 69008 Lyon, France

Correspondance : lars-petter.jordheim@univ-lyon1.fr (L.P. Jordheim)

Table of content:

- Table S1. Overall structure of NRG1 proteins based on *NRG1* coding sequences and protein sequences from databases Page 2
- Table S2. Reported *NRG1* fusions in cancer patients by May 2025. Page 4
- Figure S1. Distribution of partner genes in lung, pancreatic, gynecological, urinary and breast cancer patients Page 15
- Figure S2. Tissue distribution of *CD74*, *SLC3A2*, *SDC4*, *ATP1B1*, *CLU*, *ADAM9*, *CDH1* and *NOTCH2* fusions in reported cancer patients Page 16
- References for supplemental information Page 17

Table S1**.** Overall structure of NRG1 proteins based on *NRG1* coding sequences and protein sequences from databases (NCBI and Ensemble). 1a-13b: exon numbers; AA: amino-acids of corresponding protein; ①: cleavage site by ADAM17; ②: cleavage site by BACE1; ③: cleavage site by ADAM10; ④: slightly shorter exon with a start codon for the protein (no upstream exon); ⑤: no following exon, these exons are slightly longer than others and include a stop codon; ⑥: Stop codon within the exon due to unusual previous exon inducing modified reading frame of the last exon; ⑦: transmembrane domain with cleavage site by SPPL2a/2b; ⑧: transmembrane domain with cleavage site by γ-secretase; exon 1d: first exon of type III isoforms reported elsewhere; exons 6+7a: EGF-like domain of NRG1α isoforms; exons 6+7b: EGF-like domains of NRG1β isoforms.

| NCBI (*Ensemble*) Exon | 1a | 1b | 1c | 2 | 3 | 4 | 5 | 1d | 1e | 6 | 7a | 7b | 8 | 9 | 10 | 11 | 12 | 13a | 13b | AA |
| --- | --- | --- | --- | --- | --- | --- | --- | --- | --- | --- | --- | --- | --- | --- | --- | --- | --- | --- | --- | --- |
| NP_001153473 (*008*) |  |  |  |  |  |  |  |  |  | ① |  | ①③ | ② | ⑧ |  |  |  |  |  | 590 |
| NP_001153467 |  |  |  |  |  |  |  |  |  | ① |  | ①③ | ② | ⑧ |  |  |  |  |  | 607 |
| NP_001153471 |  |  |  |  |  |  |  |  |  | ① |  | ①③ | ② | ⑧ |  |  |  |  |  | 624 |
| NP_039256 (*001*) |  |  |  |  |  |  |  |  |  | ① |  | ①③⑤ |  |  |  |  |  |  |  | 422 |
| XP_054216364/XP_016868856 |  |  |  |  |  |  |  |  |  | ① |  | ①③ | ② | ⑧ |  |  |  |  |  | 826 |
| XP_054216363/XP_011542814 |  |  |  |  |  |  |  |  |  | ① |  | ①③ | ② | ⑧ |  |  |  |  |  | 860 |
| XP_054216367/XP_016868861 |  |  |  |  |  |  |  |  |  | ① |  | ①③ |  | ⑧ |  |  |  |  |  | 674 |
| XP_054216366/XP_016868860 |  |  |  |  |  |  |  |  |  | ① |  |  |  | ⑧ |  |  |  |  |  | 677 |
| NP_001153479 |  |  |  |  |  |  |  |  |  | ① |  |  |  |  |  |  |  |  |  | 177 |
| NP_001153477 |  |  |  |  |  |  |  |  |  | ① |  | ①③⑤ |  |  |  |  |  |  |  | 207 |
| XP_016868857/XP_054216365 (*201*) |  |  |  |  |  |  |  |  |  | ① |  | ①③ | ② | ⑧ |  |  |  |  |  | 611 |
| NP_001153474 |  |  |  |  |  |  |  |  |  | ① |  |  |  |  |  |  |  |  |  | 194 |
| NP_004486 |  |  |  |  |  |  |  |  |  | ① |  |  |  |  |  |  |  |  |  | 211 |
| NP_039252 |  |  |  |  |  |  |  |  |  | ① |  | ①③⑤ |  |  |  |  |  |  |  | 241 |
| NP_001153480 (*003*) |  |  |  |  |  |  |  |  |  | ① |  | ①③ |  | ⑧ |  |  | ⑤ |  |  | 420 |
| NP_039251 (*004*) |  |  |  |  |  |  |  |  |  | ① |  | ①③ |  | ⑧ |  |  |  |  |  | 637 |
| NP_039250 (*204*) |  |  |  |  |  |  |  |  |  | ① |  | ①③ | ② | ⑧ |  |  |  |  |  | 645 |
| NP_001153476 |  |  |  |  |  |  |  |  |  | ① |  | ①③ |  | ⑧ |  |  |  |  |  | 459 |
| Unknown (*203*) |  |  |  |  |  |  |  |  |  | ① |  | ⑥ |  |  |  |  |  |  |  | 247 |
| NP_039258 (*017*) |  |  |  |  |  |  |  |  |  | ① |  |  |  | ⑧ |  |  |  |  |  | 640 |
| NP_039254 (*006*) |  |  |  |  |  |  |  |  |  | ① |  |  |  | ⑧ |  |  |  |  |  | 462 |
| NP_039253 (*011*) |  |  |  |  |  |  |  | ②⑦ |  | ① |  | ①③⑤ |  |  |  |  |  |  |  | 296 |
| NP_001309136 |  |  |  |  |  |  |  | ②⑦ |  | ① |  | ①③ | ② | ⑧ |  |  | ⑤ |  |  | 483 |
| NP_001309134 |  |  |  |  |  |  |  | ②⑦ |  | ① |  | ①③ | ② | ⑧ |  |  |  |  |  | 700 |
| NP_001309135 |  |  |  |  |  |  |  | ②⑦ |  | ① |  | ①③ | ② | ⑧ |  |  |  |  |  | 522 |
| NP_001309126 (*205*) |  |  |  |  |  |  |  |  |  | ① |  | ①③ |  | ⑧ |  |  |  |  |  | 483 |
| NP_001153468 |  |  |  |  |  |  |  |  |  | ① |  |  |  | ⑧ |  |  |  |  |  | 308 |
| NP_001309130/NP_001309131 |  |  |  |  |  |  |  |  |  |  |  |  |  | ④⑧ |  |  |  |  |  | 382 |

Table S2. Reported *NRG1* fusions in cancer patients by May 2025. The fusion details indicate the last exon of the 5’ gene (xX, most frequently the partner gene) and the first exon of the 3’ gene (xX, most frequently *NRG1*). xU indicates unknown exon for the fusion, either for the partner gene, for *NRG1* or for both. a: *NRG1* is the 5’ gene in the fusion. Frequencies might be underestimated due to lack of information in some papers. Samples from databases might be redundant as no specific identifier is reported.

| Reference | Cancer | Gene partners and fusion details | Frequency (cases/total) | Technique | Comments |
| --- | --- | --- | --- | --- | --- |
| [1] | Lung adenocarcinoma | *CD74* x6-x6 | 1/25 and 4/102 in 2 cohorts of lung adenocarcinomas | FISH and transcriptomics | No fusion detected in a total of 63 other lung cancer subtypes or in 21 samples from other cancers |
| [2] | Lung mucinous adenocarcinoma | *VAMP2* x4-x4 | 1/986 | Anchored multiplex PCR on FFPE samples | The 986 patients were not all lung cancer |
| [3] | Invasive mucinous lung adenocarcinoma | *CD74* x6-x6  *CD74* x8-x6  *SLC3A2* x5-x6 | 2/90  3/90  1/90 | RNA sequencing | Only observed within the 36 *KRAS* wt samples |
| [4] | Lung cancers | *CD74* xU-xU  *RBPMS* xU-xU  *SDC4* xU-xU  *WRN* xU-xU | 4/753 | Transcriptome (153) and publicly available data (TCGA and Korean LUAD) | Only in samples without known driver mutations |
|  | Ovarian cancer | *RAB2IL1* xU-xU | 1 | RNA sequencing in TCGA database |  |
| [5] | Lung mucinous adenocarcinoma | *CD74* x6-x6 | 1/13  0/109 of other subtypes | RNA sequencing |  |
| [6] | NSCLC | *VAMP2* x4-x5  *CD74* x6-x6 | 1/100  1/100 | RNA sequencing |  |
| [7] | Invasive mucinous lung adenocarcinoma | *SLC3A2* x4-x4  *CD74* x6-x4  *SLC3A2* x5-x4 | 13/59  3/59  1/59 | RNA sequencing | 10/16 cases were KRAS mutated |
| [8] | Invasive mucinous lung adenocarcinoma | Unknown | 1/11 | FISH | Only performed on 11 IMA patients negative for other drivers |
| [9] | Lung cancer | *KIF13B* x2-x2 | 1/1 |  | Fusion only detected in metastasis, not in primary tumor |
| [10] | Lung adenocarcinoma | *SDC4* x2-x4 | 1/1 | RNA sequencing |  |
|  | Cholangiocarcinoma | *ATP1B1* x2-x2 | 1/1 |  |  |
| [11] | Invasive mucinous lung adenocarcinoma | *CD74* xU-xU | 1/1 | Next generation sequencing solid fusion assay and FISH |  |
| [12] | Invasive mucinous lung adenocarcinoma | *CD74* x6-x6  Unknown | 4/51  13/51 | FISH and RNA sequencing | *NRG1* rearrangements in 16/51 IMA cases and in 1/34 non-IMA cases  Only 4 FISH-positive IMA samples were analyzed by RNA sequencing, the frequency can therefore be higher |
| [13] | Invasive mucinous lung adenocarcinoma | *SLC3A2* x4-x6 | 2/2 | RNA Sequencing | Clinical response with anti-HER3 and anti-EGFR treatment |
| [14] | Lung cancer | *CD74* x6-x6  *CD74* x8-x6  *CD74* x7-x6  *SDC4* x2-x6 | 4/4 | Targeted RNA sequencing |  |
|  | Multiple cancers in MSK-IMPACT dataset | *CD74* x6-x6  *CD74* x7-x6  *CD74* x8-x6  *CD74* x7-x2  *CD74* x6-x3  *SDC4* x2-x4  *ROCK1* x2-x2  *FOXA1* x2-x2  Unknown | 1/17485  1/17485  1/17485  1/17485  1/17485  1/17485  1/17485  1/17485  2/17485 | Next-generation DNA sequencing | 7 lung adenocarcinomas  2 pancreatic adenocarcinomas  1 breast carcinoma |
|  | Multiple cancers in TGCA dataset | *AKAP13* x5-x2  *ATP1B1* x3-x2  *THAP7* x3-x2  *THBS1* x6-x6  *RAB3IL1* x9-x6  *SDC4* x4-x6  *SMAD4* x1-x6  *PDE7A* x3-x6  *PCM1* x2-x9  *STMN2* x3-x2^a^  *PMEPA1* x1-x2^a^ | 1/8984  1/8984  1/8984  1/8984  1/8984  1/8984  1/8984  1/8984  1/8984  1/8984  1/8984 |  | Breast cancer  Pancreatic cancer  Lung cancer  Head and neck cancer  Ovarian cancer  Lung cancer  Lung cancer  Head and neck cancer  Renal cancer  Prostate cancer  Uterine sarcoma  For *PCM1*-*NRG1*, no EGF-like domain is predicted  For *STMN2* and *PMEPA1*, *NRG1* is the 5’ partner in the fusion, and do not contain EGF-like domain |
|  | Ovarian cancer | *CLU* x2-x6 | 1/1 |  | Determined on a PDX |
| [15] | Pancreatic ductal adenocarcinoma | *ATP1B1* x4-x2  *SARAF-CDH6* x1-x6/7-x5  *APP* x9-x6/7-x10 | 3/17 | Whole genome sequencing and RNA sequencing | EGF-like domain (x6 and x7) is between exon 1 of *SARAF* and exon 5 of *CDH6*  EGF-like domain (x6 and x7) is inserted within the APP gene between exons 9 and 10 |
| [16] | Lung cancer | *RALGAPA1* x20-x6 | 1/1 | NGS |  |
| [17] | PDAC | *APP* x15-x6/7-x16  *ATP1B1* x4-x6  *ATP1B1* x3-x2/8-x4 | 1/47  1/47  2/47 |  | EGF-like domain is inserted within the *APP* gene between exons 15 and 16  EGF-like domain is inserted within the *ATP1B1* gene between exons 3 and 4 |
|  | Various cancers | *ATP1B1* x3-x2  *ATP1B1* x3-xU  *CDK1* x2-x6  *PDE7A* x3-x6  *SDC4* x4-x6 | 1  1  1  1  2 |  | Cholangiocarcinoma, PDAC, bladder urothelial carcinoma, head and neck squamous cell carcinoma, lung adenocarcinoma |
| [18] | Lung adenocarcinoma | *CD74* xU-xU  *ITGB1* x2-x6  *RBPMS* x5-x2 | 6/1681  1/1681  1/1684 |  |  |
| [19] | Multiple solid tumors | *CD74* x6-x6  *CD74* x8-x6  *SDC4* x2-x6  *ATB1B1* x3-x2  *ATP1B1* x2-x1  *SLC3A2* x5-x6  *TNC* x10-x6  *TSHZ2* x1-x6  *NOTCH2* x4-x6  *RBPMS* x5-x6  *MDK* x4-x6  *HMBOX1* x1-x6  *MRPL13* x2-x2  *CDH1* x3-x2  *DIP2B* x1-x2  *SETD4* x1-x2  *ADAM9* x17-x2  *RBPMS* x5-x2  *GDF15* x1-x2  *PARP8* x1-x2  *ZMYM2* x1-x2  *COX10-AS1* x1-x2  *POMK* x2-x2  *WHSC1L1* x1-x2  *VTCN1* x2-x4  *ROCK1* x1-x2  *DPSYL2* x7-x5 | 7/21858  5/21858  3/21858  2/21858  2/21858  1/21858  1/21858  1/21858  1/21858  1/21858  1/21858  1/21858  1/21858  1/21858  1/21858  1/21858  1/21858  1/21858  1/21858  1/21858  1/21858  1/21858  1/21858  1/21858  1/21858  1/21858  1/21858 |  | Total of 41 cases, lung (25), ovary (3), breast (2), GI (7), GU (2) others (2)  Fusions with *ROCK1*, *DPYSL2* and *HMBOX1* were reported out of frame variants of unknown significance |
| [20] | Lung cancer | *CD74* xU-xU  *SDC4* xU-xU  Unknown | 2/5  2/5  1/5 | Nanostring, Oncomine or RNA sequencing |  |
|  | Gastrointestinal cancer | *POMK* xU-xU | 1/1 | Caris profiling |  |
| [21] | Breast cancer | *WRN* x18-x2  *FAM91A1* x8-x2  *ARGHEF39* x2-x2  *ZNF704* x2-x3 | 1/571  1/571  1/571  1/571 |  | Fusions with *WRN*, *ARGHEF39* and *ZNF704* seem to be out of frame |
| [22] | Lung adenocarcinoma | *SLC3A2* | 1/1 |  |  |
| [23] | Lung cancer | *CD74* x6-x2 | 1/1 | NGS |  |
| [24] | Solid tumors | *VAMP2* xU-x4  *SDC4* xU-x6  *CD74* xU-x6  *UNC5D* xU-x2  *TNC* xU-x6 | 1/3263  2/3263  2/3263  1/3263  1/3263 | Targeted RNA sequencing | Lung (5 cases), prostate (1 case) and kidney (1 case) cancers |
| [25] | Lung cancer | *SLC3A2* x5-x4  *CD74* x6-x6  *CD74* xU-x5  *CD74* x7-x6  *SDC4* x2-x5  *CD74* x8-x6  *SLC3A2* x5-x6  *CADM1* x6-x6  *CD74* x5-x2  *CD74* x6-x3  *CD74* x6-x5  *F11R* x1-x6  *FGFR1* x18-x1  *FLYWCH1* x6-x2  *KRAS* x5-x1  *MDK* x6-xU  *MRPL13* x2-x2  *SDC4* x2-x6  *SDC4* x2-x4  *SLC3A2* x2-x2  *SLC3A2* x4-x6  *SLC3A2* x6-x5  *SLC3A2* xU-x6  *TNC* x10-x5  *VAMP2* x4-x4  *VAPB* x1-x2  *ATP1B1* xU-xU  *CD74* xU-xU  *DIP2B* xU-xU  *FGFR1* xU-xU  *ITGB1* xU-xU  *PLCG2* xU-xU  *RBPMS* xU-xU  *SDC4* xU-xU  *SLC3A2* xU-xU  Unknown | 15/110  12/110  11/110  3/110  3/110  2/110  2/110  1/110  1/110  1/110  1/110  1/110  1/110  1/110  1/110  1/110  1/110  1/110  1/110  1/110  1/110  1/110  1/110  1/110  1/110  1/110  1/110  14/110  1/110  2/110  1/110  1/110  1/110  1/110  1/110  20/110 | Various |  |
| [26] | Ovarian cancer | *CLU* x2-x2 | 1/1 |  | Treated with gemcitabine/erlotinib, then afatinib/gemcitabine, then trastuzumab/pertuzumab based on PDO-sensitivity |
| [27] | Lung adenocarcinoma | *CD74* xU-xU | 1/1 |  |  |
| [28] | Sarcoma | *PPHLN1* x1-x2  *HMBOX1* x1-x2  *MTUS1* x1-X6 | 1/1  1/1  1/1 | Targeted RNA NGS assay |  |
| [29] | Breast cancer | *SLC3A2* x5-x6 | 1/1 |  |  |
| [30] | Pancreatic adenocarcinoma | Unknown | 3/233 | NGS | Only *KRAS* wt tumors |
| [31] | Pancreatic cancer | *ATP1B1* x2-x2 | 2/2 | RNA-sequencing |  |
|  | NSCLC | *CD74* x7-x6 | 1/1 | RNA-sequencing |  |
| [32] | Pancreatic adenocarcinoma | *ATP1B1* xU-xU  *APP* xU-xU | 2/62  1/62 | RNA sequencing | Only in *KRAS* wt tumors |
| [33] | Pancreatic ductal adenocarcinoma | *ATP1B1* x2-x2 | 1/1 |  | The tumor is *KRAS* wt |
| [34] | Cholangiocarcinoma | *AGRN* x2-x2 | 1/1 |  | This is the only pediatric case reported (16 years old) |
| [35] | Lung adenocarcinoma | *NPTN* x1-x6 | 1/1 | NGS |  |
| [36] | Renal cell carcinoma | *KAT6A* x3-x2 | 1/1 | RNA sequencing |  |
| [37] | Solid tumors | *CD74* xU-xU  *SLC3A2* xU-xU  *ATP1B1* xU-xU  *CDH1* xU-xU  *CLU* xU-xU  *CRADD* xU-xU  *FUT10* xU-xU  *INCENP* xU-xU  *KIF22* xU-xU  *RBPMS* xU-xU  *SLC20A2* xU-xU  *VWA8* xU-xU  *XKR6* xU-xU | 8/8148  3/8148  1/8148  1/8148  1/8148  1/8148  1/8148  1/8148  1/8148  1/8148  1/8148  1/8148  1/8148 | Targeted DNA and RNA sequencing | 13 lung cancers  3 cancers of the pancreaticobiliary tract  2 gastrointestinal cancers  2 ovarian cancers  1 breast cancer  1 soft tissue sarcoma |
| [38] | Solid tumors | *CD74* xU-xU  *CDH1* xU-xU  *SDC4* xU-xU  *NOTCH2* xU-xU  *ADAM9* xU-xU  *COX10-AS1* xU-xU  *HMBOX1* xU-xU  *WDR53* xU-xU  Unknown  *VTCN1* xU-xU  *ATP1B1* xU-xU  *MRPL13* xU-xU  *SETD4* xU-xU  *CDK1* xU-xU  *GFDF1* xU-xU  *VAMP2* xU-xU  *TRAF31P2* xU-xU  *SLC3A2* xU-xU  *PARP8* xU-xU  *TNC* xU-xU  *WRN* xU-xU  *ZMYM2* xU-xU | 14/110  7/110  12/110  5/110  7/110  1/110  2/110  1/110  42/110  1/110  3/110  2/110  1/110  3/110  2/110  1/110  1/110  1/110  1/110  1/110  1/110  1/110 |  | 40 NSCLC  17 PDAC  14 bladder cancers  12 cholangiocarcinomas  6 sarcomas  7 renal cell cancers  6 colorectal cancers  4 ovarian cancers  3 breast cancers  1 neuroendocrine carcinoma |
| [39] | Cholangiocarcinoma | *SDC4* xU-xU | 1/1 |  |  |
| [40] | Serous fallopian tube carcinoma | *MYH10* x3-x2 | 1/1 |  |  |
|  | Gynecological serous carcinomas or clear cell carcinoma | *CLU* x2-x6  *CLU* x2-x2  *SARAF* x1-x2  *SCAF4* x18-x2  *WFDC2* x2-x2  *NOTCH2* x12-x6  *MUC16* x1-x2  *APP* x6-x6  *SPIDR* x16-x2  *HGSNAT* x1-x6  *SPINT2* x5-x2  *RBPMS* x5-x2  *JAG1* x13-x6  *NRP2* x15-x6  *CHMP4C* x1-x6  *CXADR* x1-x2  *TMEM65* x1-x2  *INSR* x14-x6  *ADAM9* x1-x6  *SPON1* x12-x6  *LDLR* x2-x6  *TNFRSF12A* x1-x6 | 3/14395  1/14395  2/14395  1/14395  1/14395  1/14395  1/14395  1/14395  1/14395  1/14395  1/14395  1/14395  1/14395  1/14395  1/14395  1/14395  1/14395  1/14395  1/14395  1/14395  1/14395  1/14395 |  |  |
| [41] | Various solid tumors | *CD74* xU-xU  *CD74* xU-xU^a^  *SDC4* xU-xU  *SDC4* xU-xU  *SLC3A2* xU-xU  *ATP1B1* xU-xU  *RBPMS* xU-xU  *VTCN1* xU-xU  *APP* xU-xU  *NOTCH2* xU-xU  *CDH1* xU-xU^a^  *CD44* xU-xU  *SLC4A4* xU-xU  *ASPH* xU-xU  *ADAMTSL3* xU-xU  *AGRN* xU-xU  *ALB* xU-xU  *CCT6B* xU-xU  *CFH* xU-xU  *CSMD1* xU-xU  *CXADR* xU-xU  *DAAM1* xU-xU  *DLGAP2* xU-xU  *DSCAML1* xU-xU  *F11R* xU-xU  *FBLN2* xU-xU  *FRY* xU-xU  *FUT10* xU-xU  *GTF2E2* xU-xU  *ITGB1* xU-xU  *MTUS1* xU-xU  *NSD3* xU-xU  *OLFM4* xU-xU  *POMK* xU-xU  *PTN* xU-xU  *PVALB* xU-xU  *SHANK2* xU-xU  *SLC20A2* xU-xU  *SLC34A2* xU-xU  *SOX6* xU-xU  *SPIDR* xU-xU  *ST14* xU-xU  *THAP1* xU-xU  *THBS1* xU-xU  *TNC* xU-xU  *TNFRSF10D* xU-xU  *TNFSF15* xU-xU  *VAMP2* xU-xU  *WBP2* xU-xU  *WHSC1L1* xU-xU  *WRN* xU-xU  *ZFAT* xU-xU | 71/204  1/204  11/204  1/204  29/204  23/204  5/204  5/204  4/204  4/204  4/204  3/204  3/204  2/204  1/204  1/204  1/204  1/204  1/204  1/204  1/204  1/204  1/204  1/204  1/204  1/204  1/204  1/204  1/204  1/204  1/204  1/204  1/204  1/204  1/204  1/204  1/204  1/204  1/204  1/204  1/204  1/204  1/204  1/204  1/204  1/204  1/204  1/204  1/204  1/204  1/204  1/204 | DNA- or RNA-based sequencing | 118 NSCLC, 42 PDAC, 17 cholangiocarcinomas, 10 breast cancers, 9 colorectal adenocarcinomas, 2 cancers with unknown primary, 1 endometrial sarcoma, 1 esophageal cancer, 1 gastric adenocarcinoma, 1 ovarian cancer, 1 pancreatic neuroendocrine carcinoma, 1 renal cell carcinoma |
| [42] | NSCLC | *CD74* xU-xU | 1/1 | Targeted DNA sequencing, targeted RNA sequencing, NGS | Treated with afatinib |
|  | Colorectal cancer | *CD74* xU-xU  *MATN2* xU-xU | 1/2  1/2 |  |  |
|  | Pancreatic cancer | *ATP1B1* xU-xU | 1/1 |  |  |

Figure S1. Distribution of known partner genes in lung (A, n = 234), pancreatic (B, n = 32), gynecological (C, n = 43), urinary (D, n = 20) and breast (E, n = 11) cancer patients. The 19 partner genes only occurring once in lung cancers are *CADM1*, *COX10-AS1*, *DPYSL2*, *F11R*, *FLYWCH1*, *HMBOX1*, *KIF13B*, *KIF22*, *KRAS*, *NPTN*, *PARP8*, *PLCG2*, *RALGAPA1*, *ROCK1*, *SMAD4*, *THAP7*, *VAPB*, *WDR53* and *WRN*. Urinary system includes bladder, renal cells and bladder urothelial, kidney cancers. Gynecological cancers include ovary, uterine carcinosarcoma, fallopian tubes and primary peritoneal carcinomas, endometrial sarcoma, spindle cell carcinoma in uterus. Cases with unknown partner genes are not included here. Details of cases are in Table S2.


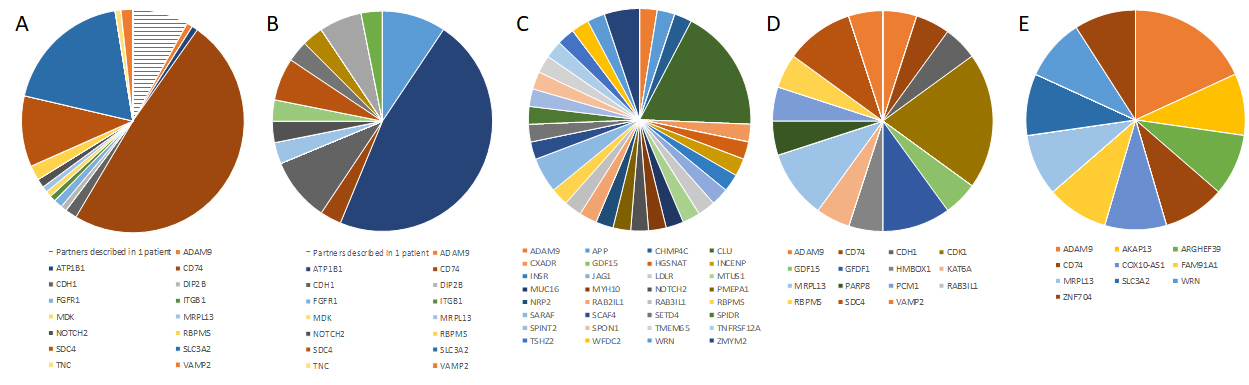


Figure S2. Tissue distribution of *CD74* (A, n = 118), *SLC3A2* (B, n = 46), *SDC4* (C, n = 32), *ATP1B1* (D, n = 23), *CLU* (E, n = 7), *ADAM9* (F, n = 9), *CDH1* (G, n = 9) and *NOTCH2* (H, n = 7) fusions in reported cancer patients with known tumor localization. Cases with unknown tumor localization are not included here. Details of cases are in Table S2.


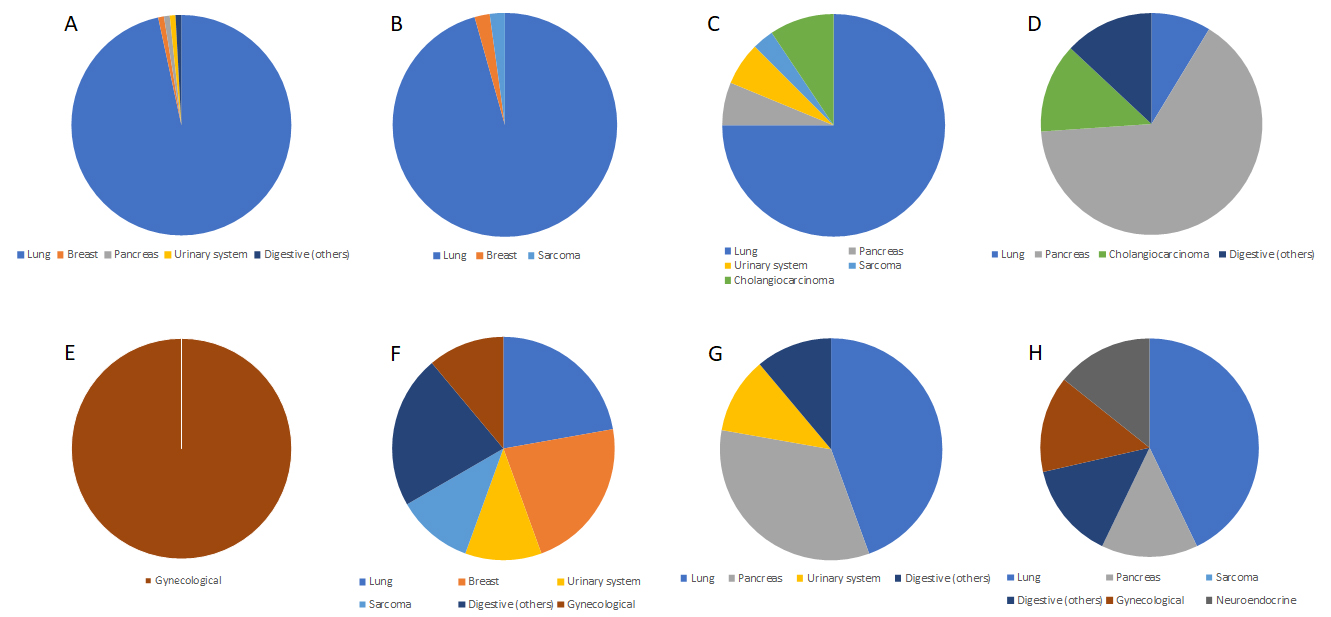


**References**

[1] Fernandez-Cuesta L, Plenker D, Osada H, Sun R, Menon R, Leenders F, Ortiz-Cuaran S, Peifer M, Bos M, Dassler J, Malchers F, Schottle J, Vogel W, Dahmen I, Koker M, Ullrich RT, Wright GM, Russell PA, Wainer Z, Solomon B, Brambilla E, Nagy-Mignotte H, Moro-Sibilot D, Brambilla CG, Lantuejoul S, Altmuller J, Becker C, Nurnberg P, Heuckmann JM, Stoelben E, Petersen I, Clement JH, Sanger J, Muscarella LA, la Torre A, Fazio VM, Lahortiga I, Perera T, Ogata S, Parade M, Brehmer D, Vingron M, Heukamp LC, Buettner R, Zander T, Wolf J, Perner S, Ansen S, Haas SA, Yatabe Y, Thomas RK. CD74-NRG1 fusions in lung adenocarcinoma. Cancer Discov, 2014; 4: 415-422. 10.1158/2159-8290.CD-13-0633.

[2] Zheng Z, Liebers M, Zhelyazkova B, Cao Y, Panditi D, Lynch KD, Chen J, Robinson HE, Shim HS, Chmielecki J, Pao W, Engelman JA, Iafrate AJ, Le LP. Anchored multiplex PCR for targeted next-generation sequencing. Nat Med, 2014; 20: 1479-1484. 10.1038/nm.3729.

[3] Nakaoku T, Tsuta K, Ichikawa H, Shiraishi K, Sakamoto H, Enari M, Furuta K, Shimada Y, Ogiwara H, Watanabe S, Nokihara H, Yasuda K, Hiramoto M, Nammo T, Ishigame T, Schetter AJ, Okayama H, Harris CC, Kim YH, Mishima M, Yokota J, Yoshida T, Kohno T. Druggable oncogene fusions in invasive mucinous lung adenocarcinoma. Clin Cancer Res, 2014; 20: 3087-3093. 10.1158/1078-0432.CCR-14-0107.

[4] Dhanasekaran SM, Balbin OA, Chen G, Nadal E, Kalyana-Sundaram S, Pan J, Veeneman B, Cao X, Malik R, Vats P, Wang R, Huang S, Zhong J, Jing X, Iyer M, Wu YM, Harms PW, Lin J, Reddy R, Brennan C, Palanisamy N, Chang AC, Truini A, Truini M, Robinson DR, Beer DG, Chinnaiyan AM. Transcriptome meta-analysis of lung cancer reveals recurrent aberrations in NRG1 and Hippo pathway genes. Nat Commun, 2014; 5: 5893. 10.1038/ncomms6893.

[5] Gow CH, Wu SG, Chang YL, Shih JY. Multidriver mutation analysis in pulmonary mucinous adenocarcinoma in Taiwan: identification of a rare CD74-NRG1 translocation case. Med Oncol, 2014; 31: 34. 10.1007/s12032-014-0034-4.

[6] Jung Y, Yong S, Kim P, Lee HY, Jung Y, Keum J, Lee S, Kim J, Kim J. VAMP2-NRG1 Fusion Gene is a Novel Oncogenic Driver of Non-Small-Cell Lung Adenocarcinoma. J Thorac Oncol, 2015; 10: 1107-1111. 10.1097/JTO.0000000000000544.

[7] Shin DH, Lee D, Hong DW, Hong SH, Hwang JA, Lee BI, You HJ, Lee GK, Kim IH, Lee YS, Han JY. Oncogenic function and clinical implications of SLC3A2-NRG1 fusion in invasive mucinous adenocarcinoma of the lung. Oncotarget, 2016; 7: 69450-69465. 10.18632/oncotarget.11913.

[8] Duruisseaux M, McLeer-Florin A, Antoine M, Alavizadeh S, Poulot V, Lacave R, Rabbe N, Cadranel J, Wislez M. NRG1 fusion in a French cohort of invasive mucinous lung adenocarcinoma. Cancer Med, 2016; 5: 3579-3585. 10.1002/cam4.838.

[9] Xia D, Le LP, Iafrate AJ, Lennerz J. KIF13B-NRG1 Gene Fusion and KRAS Amplification in a Case of Natural Progression of Lung Cancer. Int J Surg Pathol, 2017; 25: 238-240. 10.1177/1066896917693092.

[10] Jones MR, Lim H, Shen Y, Pleasance E, Ch'ng C, Reisle C, Leelakumari S, Zhao C, Yip S, Ho J, Zhong E, Ng T, Ionescu D, Schaeffer DF, Mungall AJ, Mungall KL, Zhao Y, Moore RA, Ma Y, Chia S, Ho C, Renouf DJ, Gelmon K, Jones SJM, Marra MA, Laskin J. Successful targeting of the NRG1 pathway indicates novel treatment strategy for metastatic cancer. Ann Oncol, 2017; 28: 3092-3097. 10.1093/annonc/mdx523.

[11] Cheema PK, Doherty M, Tsao MS. A Case of Invasive Mucinous Pulmonary Adenocarcinoma with a CD74-NRG1 Fusion Protein Targeted with Afatinib. J Thorac Oncol, 2017; 12: e200-e202. 10.1016/j.jtho.2017.07.033.

[12] Trombetta D, Graziano P, Scarpa A, Sparaneo A, Rossi G, Rossi A, Di Maio M, Antonello D, Mafficini A, Fabrizio FP, Manzorra MC, Balsamo T, Centra F, Simbolo M, Pantalone A, Notarangelo M, Parente P, Lucia Dimitri MC, Bonfitto A, Fiordelisi F, Storlazzi CT, L'Abbate A, Taurchini M, Maiello E, Fazio VM, Muscarella LA. Frequent NRG1 fusions in Caucasian pulmonary mucinous adenocarcinoma predicted by Phospho-ErbB3 expression. Oncotarget, 2018; 9: 9661-9671. 10.18632/oncotarget.23800.

[13] Kim HS, Han JY, Shin DH, Lim KY, Lee GK, Kim JY, Jacob W, Ceppi M, Weisser M, James I. EGFR and HER3 signaling blockade in invasive mucinous lung adenocarcinoma harboring an NRG1 fusion. Lung Cancer, 2018; 124: 71-75. 10.1016/j.lungcan.2018.07.026.

[14] Drilon A, Somwar R, Mangatt BP, Edgren H, Desmeules P, Ruusulehto A, Smith RS, Delasos L, Vojnic M, Plodkowski AJ, Sabari J, Ng K, Montecalvo J, Chang J, Tai H, Lockwood WW, Martinez V, Riely GJ, Rudin CM, Kris MG, Arcila ME, Matheny C, Benayed R, Rekhtman N, Ladanyi M, Ganji G. Response to ERBB3-Directed Targeted Therapy in NRG1-Rearranged Cancers. Cancer Discov, 2018; 8: 686-695. 10.1158/2159-8290.CD-17-1004.

[15] Heining C, Horak P, Uhrig S, Codo PL, Klink B, Hutter B, Frohlich M, Bonekamp D, Richter D, Steiger K, Penzel R, Endris V, Ehrenberg KR, Frank S, Kleinheinz K, Toprak UH, Schlesner M, Mandal R, Schulz L, Lambertz H, Fetscher S, Bitzer M, Malek NP, Horger M, Giese NA, Strobel O, Hackert T, Springfeld C, Feuerbach L, Bergmann F, Schrock E, von Kalle C, Weichert W, Scholl C, Ball CR, Stenzinger A, Brors B, Frohling S, Glimm H. NRG1 Fusions in KRAS Wild-Type Pancreatic Cancer. Cancer Discov, 2018; 8: 1087-1095. 10.1158/2159-8290.CD-18-0036.

[16] McCoach CE, Le AT, Gowan K, Jones K, Schubert L, Doak A, Estrada-Bernal A, Davies KD, Merrick DT, Bunn PA, Jr., Purcell WT, Dziadziuszko R, Varella-Garcia M, Aisner DL, Camidge DR, Doebele RC. Resistance Mechanisms to Targeted Therapies in ROS1(+) and ALK(+) Non-small Cell Lung Cancer. Clin Cancer Res, 2018; 24: 3334-3347. 10.1158/1078-0432.CCR-17-2452.

[17] Jones MR, Williamson LM, Topham JT, Lee MKC, Goytain A, Ho J, Denroche RE, Jang G, Pleasance E, Shen Y, Karasinska JM, McGhie JP, Gill S, Lim HJ, Moore MJ, Wong HL, Ng T, Yip S, Zhang W, Sadeghi S, Reisle C, Mungall AJ, Mungall KL, Moore RA, Ma Y, Knox JJ, Gallinger S, Laskin J, Marra MA, Schaeffer DF, Jones SJM, Renouf DJ. NRG1 Gene Fusions Are Recurrent, Clinically Actionable Gene Rearrangements in KRAS Wild-Type Pancreatic Ductal Adenocarcinoma. Clin Cancer Res, 2019; 25: 4674-4681. 10.1158/1078-0432.CCR-19-0191.

[18] Pan Y, Zhang Y, Ye T, Zhao Y, Gao Z, Yuan H, Zheng D, Zheng S, Li H, Li Y, Jin Y, Sun Y, Chen H. Detection of Novel NRG1, EGFR, and MET Fusions in Lung Adenocarcinomas in the Chinese Population. J Thorac Oncol, 2019; 14: 2003-2008. 10.1016/j.jtho.2019.07.022.

[19] Jonna S, Feldman RA, Swensen J, Gatalica Z, Korn WM, Borghaei H, Ma PC, Nieva JJ, Spira AI, Vanderwalde AM, Wozniak AJ, Kim ES, Liu SV. Detection of NRG1 Gene Fusions in Solid Tumors. Clin Cancer Res, 2019; 25: 4966-4972. 10.1158/1078-0432.CCR-19-0160.

[20] Cadranel J, Liu SV, Duruisseaux M, Branden E, Goto Y, Weinberg BA, Heining C, Schlenk RF, Cheema P, Jones MR, Drilon A, Trombetta D, Muscarella LA, Tolba K, Gounant V, Cseh A, Solca F, Laskin JJ, Renouf DJ. Therapeutic Potential of Afatinib in NRG1 Fusion-Driven Solid Tumors: A Case Series. Oncologist, 2021; 26: 7-16. 10.1634/theoncologist.2020-0379.

[21] Howarth KD, Mirza T, Cooke SL, Chin SF, Pole JC, Turro E, Eldridge MD, Garcia RM, Rueda OM, Boursnell C, Abraham JE, Caldas C, Edwards PAW. NRG1 fusions in breast cancer. Breast Cancer Res, 2021; 23: 3. 10.1186/s13058-020-01377-5.

[22] Odintsov I, Mattar MS, Lui AJW, Offin M, Kurzatkowski C, Delasos L, Khodos I, Asher M, Daly RM, Rekhtman N, de Stanchina E, Ganji G, Ladanyi M, Somwar R. Novel Preclinical Patient-Derived Lung Cancer Models Reveal Inhibition of HER3 and MTOR Signaling as Therapeutic Strategies for NRG1 Fusion-Positive Cancers. J Thorac Oncol, 2021; 16: 1149-1165. 10.1016/j.jtho.2021.03.013.

[23] Wu X, Zhang D, Shi M, Wang F, Li Y, Lin Q. Successful targeting of the NRG1 fusion reveals durable response to afatinib in lung adenocarcinoma: a case report. Ann Transl Med, 2021; 9: 1507. 10.21037/atm-21-3923.

[24] Ptakova N, Martinek P, Holubec L, Janovsky V, Vancurova J, Grossmann P, Navarro PA, Rodriguez Moreno JF, Alaghehbandan R, Hes O, Majek O, Pesek M, Michal M, Ondic O. Identification of tumors with NRG1 rearrangement, including a novel putative pathogenic UNC5D-NRG1 gene fusion in prostate cancer by data-drilling a de-identified tumor database. Genes Chromosomes Cancer, 2021; 60: 474-481. 10.1002/gcc.22942.

[25] Drilon A, Duruisseaux M, Han JY, Ito M, Falcon C, Yang SR, Murciano-Goroff YR, Chen H, Okada M, Molina MA, Wislez M, Brun P, Dupont C, Branden E, Rossi G, Schrock A, Ali S, Gounant V, Magne F, Blum TG, Schram AM, Monnet I, Shih JY, Sabari J, Perol M, Zhu VW, Nagasaka M, Doebele R, Camidge DR, Arcila M, Ou SI, Moro-Sibilot D, Rosell R, Muscarella LA, Liu SV, Cadranel J. Clinicopathologic Features and Response to Therapy of NRG1 Fusion-Driven Lung Cancers: The eNRGy1 Global Multicenter Registry. J Clin Oncol, 2021; 39: 2791-2802. 10.1200/JCO.20.03307.

[26] Murumagi A, Ungureanu D, Khan S, Arjama M, Valimaki K, Ianevski A, Ianevski P, Bergstrom R, Dini A, Kanerva A, Koivisto-Korander R, Tapper J, Lassus H, Loukovaara M, Magi A, Hirasawa A, Aoki D, Pietiainen V, Pellinen T, Butzow R, Aittokallio T, Kallioniemi O. Drug response profiles in patient-derived cancer cells across histological subtypes of ovarian cancer: real-time therapy tailoring for a patient with low-grade serous carcinoma. Br J Cancer, 2023; 128: 678-690. 10.1038/s41416-022-02067-z.

[27] Chen K, Li W, Xi X, Zhong J. A case of multiple primary lung adenocarcinoma with a CD74-NRG1 fusion protein and HER2 mutation benefit from combined target therapy. Thorac Cancer, 2022; 13: 3063-3067. 10.1111/1759-7714.14636.

[28] Dermawan JK, Zou Y, Antonescu CR. Neuregulin 1 (NRG1) fusion-positive high-grade spindle cell sarcoma: A distinct group of soft tissue tumors with metastatic potential. Genes Chromosomes Cancer, 2022; 61: 123-130. 10.1002/gcc.23008.

[29] Fontana E, Torga G, Fostea R, Cleator S, Wasserman E, Murat A, Arkenau HT. Sustained Tumor Regression With Zenocutuzumab, a Bispecific Antibody Targeting Human Epidermal Growth Factor Receptor 2/Human Epidermal Growth Factor Receptor 3 Signaling, in NRG1 Fusion-Positive, Estrogen Receptor-Positive Breast Cancer After Progression on a Cyclin-Dependent Kinase 4/6 Inhibitor. JCO Precis Oncol, 2022; 6: e2100446. 10.1200/PO.21.00446.

[30] Philip PA, Azar I, Xiu J, Hall MJ, Hendifar AE, Lou E, Hwang JJ, Gong J, Feldman R, Ellis M, Stafford P, Spetzler D, Khushman MM, Sohal D, Lockhart AC, Weinberg BA, El-Deiry WS, Marshall J, Shields AF, Korn WM. Molecular Characterization of KRAS Wild-type Tumors in Patients with Pancreatic Adenocarcinoma. Clin Cancer Res, 2022; 28: 2704-2714. 10.1158/1078-0432.CCR-21-3581.

[31] Schram AM, Odintsov I, Espinosa-Cotton M, Khodos I, Sisso WJ, Mattar MS, Lui AJW, Vojnic M, Shameem SH, Chauhan T, Torrisi J, Ford J, O'Connor MN, Geuijen CAW, Schackmann RCJ, Lammerts van Bueren JJ, Wasserman E, de Stanchina E, O'Reilly EM, Ladanyi M, Drilon A, Somwar R. Zenocutuzumab, a HER2xHER3 Bispecific Antibody, Is Effective Therapy for Tumors Driven by NRG1 Gene Rearrangements. Cancer Discov, 2022; 12: 1233-1247. 10.1158/2159-8290.CD-21-1119.

[32] Topham JT, Tsang ES, Karasinska JM, Metcalfe A, Ali H, Kalloger SE, Csizmok V, Williamson LM, Titmuss E, Nielsen K, Negri GL, Spencer Miko SE, Jang GH, Denroche RE, Wong HL, O'Kane GM, Moore RA, Mungall AJ, Loree JM, Notta F, Wilson JM, Bathe OF, Tang PA, Goodwin R, Morin GB, Knox JJ, Gallinger S, Laskin J, Marra MA, Jones SJM, Schaeffer DF, Renouf DJ. Integrative analysis of KRAS wildtype metastatic pancreatic ductal adenocarcinoma reveals mutation and expression-based similarities to cholangiocarcinoma. Nat Commun, 2022; 13: 5941. 10.1038/s41467-022-33718-7.

[33] Thavaneswaran S, Chan WY, Asghari R, Grady JP, Deegan M, Jansen VM, Thomas DM. Clinical Response to Seribantumab, an Anti-Human Epidermal Growth Factor Receptor-3 Immunoglobulin 2 Monoclonal Antibody, in a Patient With Metastatic Pancreatic Ductal Adenocarcinoma Harboring an NRG1 Fusion. JCO Precis Oncol, 2022; 6: e2200263. 10.1200/PO.22.00263.

[34] Mitchell SG, Basu GD, Eaton B, Goodman LJ, Goldsmith KC. Identification of a Novel NRG1 Fusion with Targeted Therapeutic Implications in Locally Advanced Pediatric Cholangiocarcinoma: A Case Report. Case Rep Oncol, 2023; 16: 249-255. 10.1159/000530164.

[35] Nie X, Zhang P, Bie Z, Song C, Zhang M, Ma D, Cui D, Cheng G, Li H, Lei Y, Su X, Wu W, Li L. Durable response to afatinib in advanced lung adenocarcinoma harboring a novel NPTN-NRG1 fusion: a case report. World J Surg Oncol, 2023; 21: 246. 10.1186/s12957-023-03129-z.

[36] Dawood A, MacMahon S, Dang MT, Tran MGB, Bex A, Boleti E, Sheikh SE. Case Report: Disease progression of renal cell carcinoma containing a novel putative pathogenic KAT6A::NRG1 fusion on Ipilimumab- Nivolumab immunotherapy. A case study and review of the literature. Front Oncol, 2023; 13: 1111706. 10.3389/fonc.2023.1111706.

[37] Cha YJ, Lee C, Joo B, Kim KA, Lee CK, Shim HS. Clinicopathological Characteristics of NRG1 Fusion-Positive Solid Tumors in Korean Patients. Cancer Res Treat, 2023; 55: 1087-1095. 10.4143/crt.2023.682.

[38] Liu SV, Frohn C, Minasi L, Fernamberg K, Klink AJ, Gajra A, Savill KMZ, Jonna S. Real-world outcomes associated with afatinib use in patients with solid tumors harboring NRG1 gene fusions. Lung Cancer, 2024; 188: 107469. 10.1016/j.lungcan.2024.107469.

[39] Wilding B, Woelflingseder L, Baum A, Chylinski K, Vainorius G, Gibson N, Waizenegger IC, Gerlach D, Augsten M, Spreitzer F, Shirai Y, Ikegami M, Tilandyova S, Scharn D, Pearson MA, Popow J, Obenauf AC, Yamamoto N, Kondo S, Opdam FL, Bruining A, Kohsaka S, Kraut N, Heymach JV, Solca F, Neumuller RA. Zongertinib (BI 1810631), an irreversible HER2 TKI, spares EGFR signaling and improves therapeutic response in preclinical models and patients with HER2-driven cancers. Cancer Discov, 2024; 10.1158/2159-8290.CD-24-0306.

[40] Crymes A, Evans MG, Adeyelu T, Reid J, Ibe IO, Oberley MJ, Tseng JH. Case report: High grade serous fallopian tube carcinoma with rare NRG1 gene fusion presenting as widespread peritoneal carcinomatosis. Front Oncol, 2024; 14: 1472725. 10.3389/fonc.2024.1472725.

[41] Schram AM, Goto K, Kim DW, Macarulla T, Hollebecque A, O'Reilly EM, Ou SI, Rodon J, Rha SY, Nishino K, Duruisseaux M, Park JO, Neuzillet C, Liu SV, Weinberg BA, Cleary JM, Calvo E, Umemoto K, Nagasaka M, Springfeld C, Bekaii-Saab T, O'Kane GM, Opdam F, Reiss KA, Joe AK, Wasserman E, Stalbovskaya V, Ford J, Adeyemi S, Jain L, Jauhari S, Drilon A, e NI. Efficacy of Zenocutuzumab in NRG1 Fusion-Positive Cancer. N Engl J Med, 2025; 392: 566-576. 10.1056/NEJMoa2405008.

[42] Rodon J, Rothe M, Mangat PK, Garrett-Mayer E, Cannon TL, Hobbs E, Kalemkerian GP, Hinshaw DC, Gregory A, Grantham GN, Halabi S, Schilsky RL. Afatinib in patients with solid tumors with neuregulin 1 (NRG1) fusions: a case series from the Targeted Agent and Profiling Utilization Registry (TAPUR) Study. ESMO Open, 2025; 10: 104545. 10.1016/j.esmoop.2025.104545.
